# A Novel Di-Leucine Motif at the N-Terminus of Human Organic Solute Transporter Beta Is Essential for Protein Association and Membrane Localization

**DOI:** 10.1371/journal.pone.0158269

**Published:** 2016-06-28

**Authors:** Shuhua Xu, Carol J. Soroka, An-Qiang Sun, Donald S. Backos, Albert Mennone, Frederick J. Suchy, James L. Boyer

**Affiliations:** 1 Department of Internal Medicine and Yale Liver Center, Yale University School of Medicine, New Haven, Connecticut, United States of America; 2 Department of Pediatrics, University of Colorado Anschutz Medical Campus, Aurora, United States of America; 3 Skaggs School of Pharmacy and Pharmaceutical Sciences, University of Colorado Anschutz Medical Campus, Aurora, United States of America; Cambridge University, UNITED KINGDOM

## Abstract

The heteromeric membrane protein Organic Solute Transporter alpha/beta is the major bile acid efflux transporter in the intestine. Physical association of its alpha and beta subunits is essential for their polarized basolateral membrane localization and function in the transport of bile acids and other organic solutes. We identified a highly conserved acidic dileucine motif (-EL_20_L_21_EE) at the extracellular amino-tail of organic solute transporter beta from multiple species. To characterize the role of this protein interacting domain in the association of the human beta and alpha subunits and in membrane localization of the transporter, Leu20 and Leu21 on the amino-tail of human organic solute transporter beta were replaced with alanines by site-directed mutagenesis. Co-immunoprecipitation study in HEK293 cells demonstrated that substitution of the leucine residues with alanines prevented the interaction of the human beta mutant with the alpha subunit. Membrane biotinylation demonstrated that the LL/AA mutant eliminated membrane expression of both subunits. Computational-based modelling of human organic solute transporter beta suggested that the LL/AA mutation substantially alters both the structure and lipophilicity of the surface, thereby not only affecting the interaction with the alpha subunit but also possibly impacting the capacity of the beta subunit to traffick through the cell and interact with the membrane. In summary, our findings indicate that the dileucine motif in the extracellular N-terminal region of human organic solute transporter beta subunit plays a critical role in the association with the alpha subunit and in its polarized plasma membrane localization.

## Introduction

Organic Solute Transporter beta (OSTβ, SLC51b) is associated with Organic Solute Transporter alpha (OSTα, SLC51a) subunit to form a novel heteromeric transporter (OSTα-OSTβ) that was discovered in 2003 [1]. These two proteins are co-expressed at the basolateral membranes in the ileum, kidney, and liver, as well as other epithelia, and function together to transport bile acids and other important sterol-derived molecules via a sodium-independent mechanism characterized by facilitated diffusion [2–4]. Human OSTβ (hOSTβ) contains 128 amino acids and has a predicted structure of one transmembrane (TM) domain by hydropathy analysis [5]. Human OSTα contains 340 amino acids and has seven transmembrane domains. Human OSTα and mouse Ostα share 83% amino acid identity with each other and 41% amino acid identity with skate Ostα, the species from which it was originally cloned. Human OSTβ shares 63% amino acid identity with mouse Ostβ and 25% with skate Ostβ [6]. Despite this relatively low amino acid identity between human and skate, Seward et al [1] showed that subunits from different species could functionally complement each other, suggesting conserved domains between species. Previous studies [7] demonstrated that the interaction between Ostα and Ostβ was required not only for delivery of the proteins to the plasma membrane, but for the stability of the heteromeric transporter. In Ostα -/- mice, Ostβ mRNA levels were maintained, yet Ostβ protein was not detectable, indicating that Ostβ protein is not stable in the absence of Ostα. Further, co-expression of both Ostα and Ostβ was required to convert the Ostα subunit to a mature glycosylated endoglycosidase H-resistant form, suggesting that co-expression facilitates the trafficking of Ostα through the Golgi apparatus. Immunolocalization studies showed that co-expression was necessary for plasma membrane expression of both Ostα and Ostβ [2]. Thus, it is the presence and interaction of the two subunits that is critical to the stability of the heteromeric, intact transporter, not the glycosylation state of the alpha subunit [8]. Other studies [7,9] demonstrated that co-expression of OSTα and β, but not the individual subunits, stimulated Na+- independent bile acid uptake and the apical-to basolateral transport of ^3^H-taurocholate. However, the mechanisms involved in the polarized membrane trafficking of OST proteins are not fully understood. It remains to be determined which domain of OSTβ is required for the formation of a functional heterodimer with OSTα and which amino acids affect this interaction [7].

Previous studies have suggested that the N-terminal domain of OSTβ is important in the interaction of the two subunits and in the functional activity of the transporter [10,11]. Therefore, we performed a systematic screen for protein interaction and targeting motifs within the N-terminal domain of OSTβ. By combining alignments of OSTβ/Ostβ sequences from different species with protein structure analysis and mutagenesis approaches, we determined that the leucine containing motifs within the OSTβ extracellular N-terminus play a critical role in regulating OST protein biogenesis, OSTα-β heterodimerization, and the membrane localization of this heteromeric organic solute transporter.

## Materials and Methods

### Antibodies and reagents

Rabbit polyclonal to Myc antibody was purchased from Abcam (Cambridge, MA), monoclonal anti-Flag M2 antibody was from Sigma (St Louis, MO, USA), and Living Colore A.V. monoclonal anti-GFP antibody was from Clontech (Mountain View, CA, USA). Anti-Halo Tag monoclonal antibody and HaloTag TMR Ligand were purchased from Promega (Madison, WI, USA). Cell-culture supplies were obtained from Life Technologies, Inc. (Rockville, MD). Subcloning reagents, restriction enzymes and competent cells were obtained from Stratagene (La Jolla, CA), GIBCO BRL (Gaithersburg, MD), New England BioLabs (Beverly, MA), and Invitrogen (Carlsbad, CA).

### DNA constructs

The pcDNA3.1 C-terminal Flag-tagged wild-type human OSTα and the pcDNA3 C-terminal Myc-Tagged wild-type human OSTβ have been described earlier [8]. The pcDNA GFP-tagged OSTα has been described [11]. PCR-based mutagenesis was used to generate the OSTβ mutant constructs. The N-terminal sequences L20L21 were replaced by alanines by using the QuickChange Site Directed Mutagenesis Kit (Stratagene, la Jolla, CA). The pHTC-HaloTag vector was kindly provided by Promega. A full-length wild type OSTβ or a full-length LL/AA mutant OSTβ was amplified from pcDNA Myc-tagged OSTβ and subcloned into the pHTC-HaloTag vector at the sites of EcoR1/Xho1. The primers for PCR amplification are as followings; pHTC-OSTβ forward, gatcgcttccgaattcgccaccatgagcacagtgaggg, pHTC-OSTβ reverse, cagtggttggctcgaggctctcatttctggtacatcc. The sequences of the DNA constructs were verified by W. M. Keck DNA sequencing facility at Yale University.

### Cell culture and transfection

HEK293 cells were purchased from the American Type Culture Collection (Manassas, VA) and grown in Dulbecco’s modified Eagle’s high glucose medium (Invitrogen, Carlsbad, CA, USA) containing 10% fetal bovine serum (FBS) and 1% penicillin-streptomycin. Cells were cultured at 37°C in a 5% CO_2_ atmosphere incubator. HEK293 cells were transfected using Fugene HD transfection reagent (Roche Diagnostics, Indianapolis, IN, USA) or using Lipofectamine 2000 transfection reagent (Invitrogen), both according to manufacturer’s instructions.

### RNA expression and cDNA amplification

Total RNA was extracted from the transfected HEK293 cells with OSTα and wild-type or LL/AA mutant OSTβ constructs by TRIzol reagent. Equal amounts of RNA were used for the cDNA synthesis by SuperScript Reverse Transcriptase reagent according to the manufacturer (Invitrogen). mRNA levels of the transfected constructs were quantified by RT-PCR. The primer sequences for this study were as follows: β-actin, cgtcttcccctccatcg (forward) and ctcgttaatgtcacgcac (reverse); OSTα, ctgaagaccaattacggcatc (forward) and gagggcaagttccacagg (reverse). PCR reactions were carried out for 35 cycles (98°C for 25 sec, 55°C for 25 sec, 72°C for 1 min) based on the instruction of Taq98 Hot Start Master Mix from Lucigen Corporation (Middleton, WI).

### Coimmunoprecipitation

HEK293 cells were plated in 6-well plates and transfected with the various constructs. After 24–48 hrs, the cells were lysed in M-PER Mammalian Protein Extraction Reagent (Thermo Scientific, Rockford, IL) containing protease inhibitors (Roche Diagnostics) on ice for 30 min and then centrifuged at 16,000xg for 15 min. Cleared supernatants were incubated with primary antibodies overnight at 4°C and then with protein A/G beads for 2–3 hours. The beads were washed three times in lysis buffer and incubated in Laemmli sample buffer (BioRad) for 10 min at RT or heated at 70°C for 10 min. Bound immune complexes were analyzed by SDS-PAGE and immunoblotting.

### Cell surface biotinylation

Cell surface biotinylation of OST alpha and beta was performed as described [12]. HEK293 cells were grown on poly-lysine-coated 6-well plates and transciently transfected with OSTα-Flag and OSTβ-Myc wild type or mutant constructs individually or together. The cells were biotinylated after 24–48 h transfection. After biotinylation, the cells were lysed immediately with M-PER lysis buffer containing protease inhibitor cocktail. Then equal amount of protein in the cell lysates was incubated with Streptavidin-agarose beads to isolate the biotinylated cell membrane proteins. The OSTα-flag or OSTβ-Myc protein was detected in the pool of surface proteins by electrophoresis and immunoblotting using anti-myc antibody (1:1000) and anti-flag antibody (1:1000).

### Immunoblotting

Protein samples were separated on 12% SDS-PAGE gels and transferred to nitrocellulose membranes (Perkin Elmer, Woodbridge, ON, Canada). Membranes were blocked in Tris-buffered saline containing 0.1% Tween 20 and 5% nonfat dry milk and incubated with primary antibodies for 2h at RT or overnight at 4°C and then with horseradish peroxidase-conjugated anti-rabbit or anti-mouse IgG (Sigma) and enhanced chemiluminescence detection (Pierce Chemical, Rockford, IL, USA).

### Immunofluorescence

HEK293 cells were plated on poly-lysine coated coverslips. Twenty four hours after transfection, the cells were labeled by replacing the existing volume of medium with the medium containing 5μM HaloTag TMR ligand. Cells were incubated for 30 min in a 37°C + CO_2_ cell culture incubator, then the ligand-containing medium was gently replaced with an equal volume of warm fresh 1 x PBS. This was repeated two times to wash out unbound ligand. The cells were fixed with an equal volume of warm 4% paraformaldehyde/0.5mM sucrose /1 x PBS (pH7.5) and incubated for 15 min at room temperature. The fixative was replaced with an equal volume of 1 x PBS, mounted on a microscope slide with Hard mounting medium containing DAPI (Vector Labs) and then transfered to a microscope (Leica SP5) to capture images.

### Proteasomes and lysosome pathway

OSTα-flag was co-transfected with WT or LL/AA Mut OSTβ-Myc in HEK293 cells. Transfected cells were treated with MG132 (10μM) or (vehicle) for 24 h, or with a combination of lysosome inhibitors, leupeptin (50μg/ml) / pepstatin A (4μg/ml) or vehicle for 16 hours. Cell lysates were subject to SDS-PAGE and immunoblotting.

### Molecular modeling

All molecular modeling studies were conducted using Accelrys Discovery Studio 3.1 (Accelrys Software, Inc., San Diego, CA; http://accelrys.com). All crystal structure coordinates used in these studies were obtained from the protein data bank (http://www.pdb.org). The protein homology model of human OSTβ was constructed with the MODELLER protocol [13] using the crystal structures of acetyl CoA synthetase [14] (PDB ID: 1PG4) and leucyl-tRNA synthetase [15] (PDB ID: 1WKB) as templates. Prediction of the orientation of the OSTβ homology model in the plasma membrane was calculated using the Generalized-Born with implicit membrane algorithm [16]. The transmembrane segment of the model was then subjected to conformational optimization utilizing the LOOPER algorithm [17] followed by a final energy minimization with the conjugate gradient minimization protocol (10,000 iterations with a root mean square cutoff of 0.01 kcal/mol) using a CHARMm forcefield and the Generalized Born implicit solvent model [18].

## Results

### N-Terminus of OSTβ contains a cluster of acidic dileucine-based motifs

**Fig 1:**
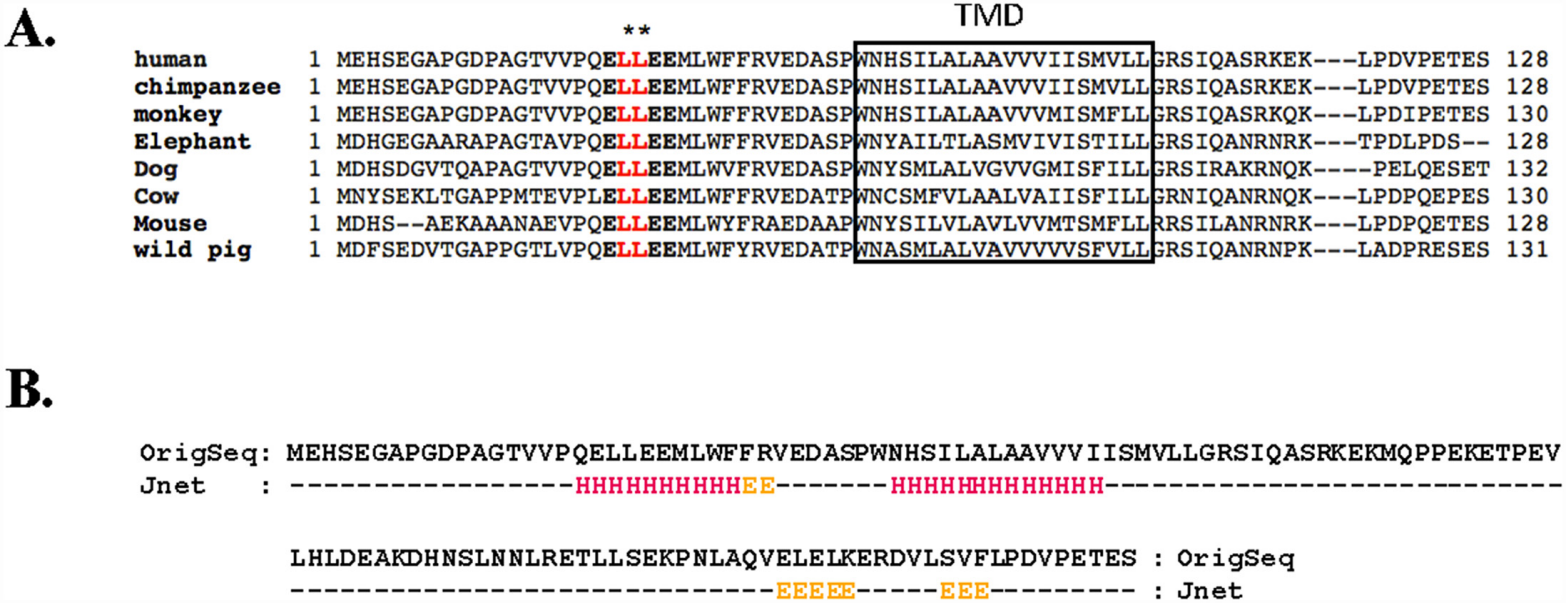
Identification and prediction of putative leucine-based motifs in the N-terminal tail of OSTβ. (**A**) Sequence of alignment of full-length OSTβ from different species. A highly conserved acidic dileucine motif (ELLEE) was identified within the N-terminal region of all species. The stars “**” indicate a dileucine motif at position L20L21. The predicted TM domain is boxed. “-” denotes that the part of the sequence is skipped. (**B**) Prediction of the location of secondary structure at OSTβ extracellular and transmembrane (TM) domains. The prediction classifies each amino acid residue as belonging to alpha helix ('H') or beta sheet ('E') using a neural network called Jnet.

Previous immunoprecipitation (IP) studies [11] have shown that the deletion of the extracellular domain of OSTβ disrupted the interaction of OSTβ with OSTα, suggesting that the extracellular domain of OSTβ contains crucial motifs for OSTα and β protein-protein interaction. A sequence alignment of the N-terminal extracellular domain of OSTβ from 8 different species showed the presence of highly conserved consensus leucine-based motifs (Fig 1A). The N-terminal tail encompassing residues 1–35 contains a putative dileucine-based motif at position L20L21 in all of the species. The predicted secondary protein structure for the OSTβ using Jnet, a web server for secondary structure prediction, is shown in Fig 1B, indicating that the dileucine motif falls within a region of alpha helix, potential residues for protein interaction.

### N-terminal dileucine motif affects OSTα and β association and localization

**Fig 2:**
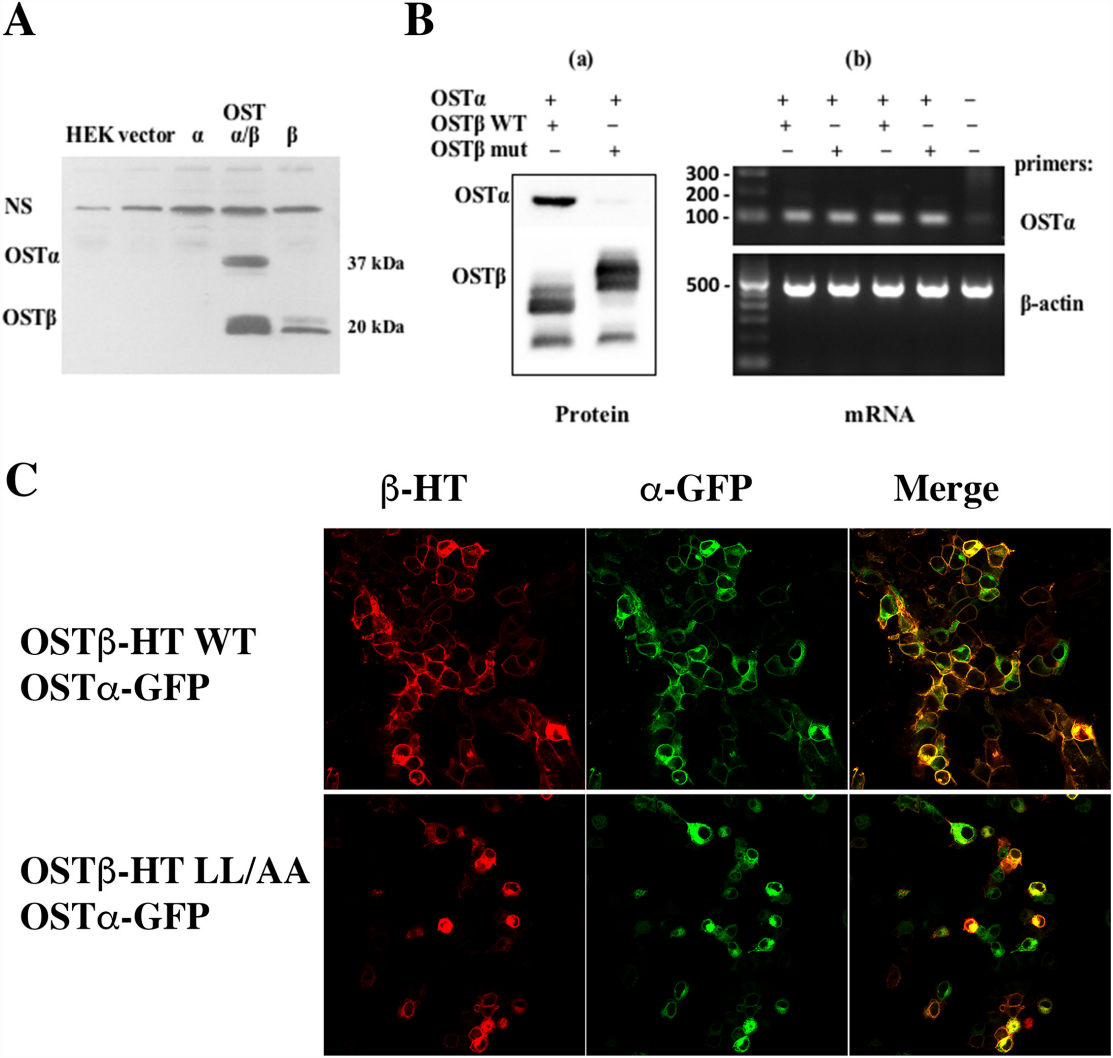
Mutation in the N-terminal dileucine residues affects the protein expression and localization. (**A**) Co-expression of both the alpha and beta subunits is required for expression of the heteromeric transporter. OSTα or OSTβ construct was transfected individually or together in HEK293 cells. Untransfected cells and transfected pcDNA3 vector cells are used as background controls. (**B-a**) Mutation of OSTβ dileucine residues greatly decreased the OSTα total protein expression, indicating that the acidic dileucine motifs at the N-terminus of the OSTβ might play an important role in the stability of the heteromeric transporter which depends upon the interaction of the two subunits. (**B-b**) mRNA levels of transfected OSTα with wild-type or mutant OSTβ constructs in HEK293 cells was not affected by the LL/AA mutation. Fig 2B is representative of two individual experiments. (**C**) Immunofluorescence imaging of transfected HEK293 cells shows that co-expression of WT alpha and beta resulted in plasma membrane localization of both subunits (***a***). However, co-transfection of WT alpha and LL/AA OSTβ resulted largely in intracellular retention of the subunits (***b***).

We first confirmed by PAGE that co-expression of both the alpha and beta subunits was required for expression of the WT heteromeric transporter in HEK293 cells (Fig 2A). Site-directed mutageneses was then used to substitute A20A21 for L20L21 into the extracellular domain of OSTβ. Mutation of the dileucine motif greatly reduced OSTα expression in total cell extracts after transfection in HEK293 cells (Fig 2B *on left panel*). Although the expression level of the LL/AA OSTβ mutant was similar to the WT OSTβ, little OSTα was detected in the total cell lysate, suggesting that the dileucine motif at the N-terminus of OSTβ might play an important role in stability of the OSTα subunit. Interestingly, LL/AA OSTβ mutant migrated at slower rate in SDS-PAGE. Because the molecular weight would not be changed by the mutation, this suggested that the change resulted in a secondary structure that was not completely denatured by the SDS, allowing for an aberrant migratory rate in the gel [19].

To verify that the reduced OSTα protein level is not due to an effect on its mRNA expression, HEK293 cells were transfected with WT OSTα and either WT OSTβ or LL/AA OSTβ mutant. RT-PCR confirmed that mRNA levels of OSTα were the same with both constructs of OSTβ (Fig 2B *on right panel*). Immunofluorescent imaging of the transfected HEK293 cells showed that the mutation of the dileucine motif in the OSTβ subunit prevented the plasma membrane localization of the heteromeric transporter (Fig 2C).

### Mutation in the N-terminal dileucine residues affects the interaction of the subunits

**Fig 3:**
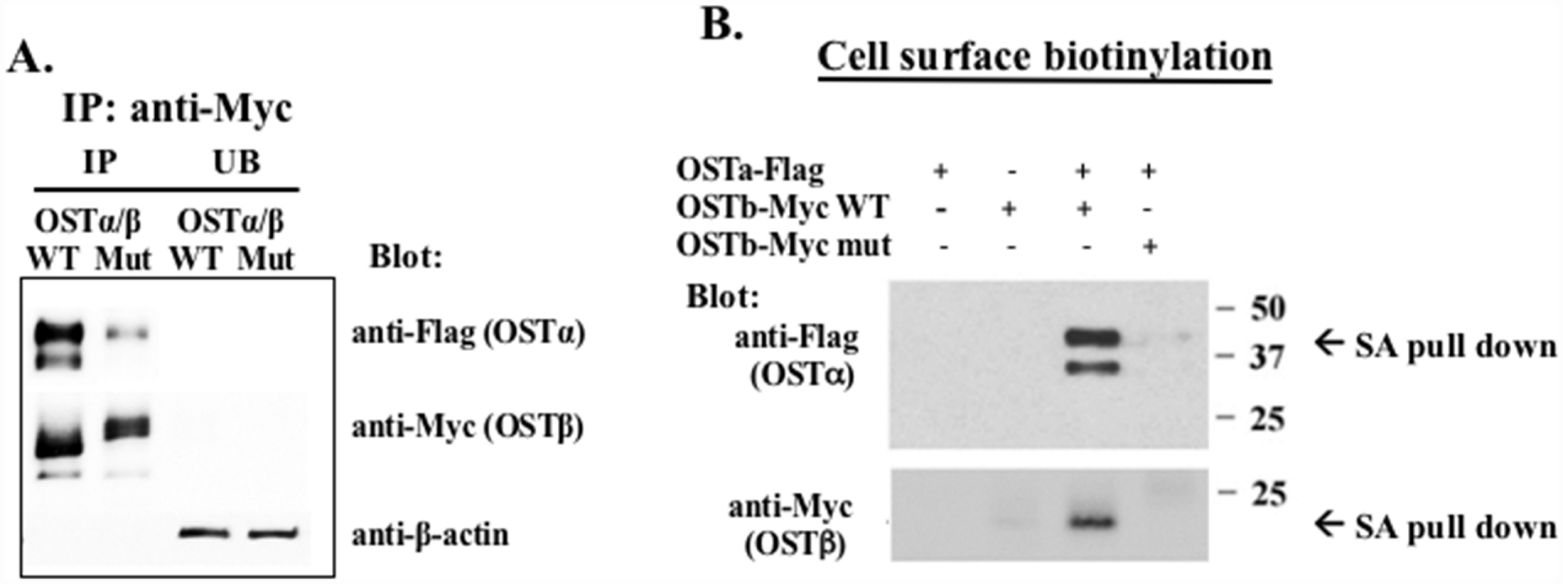
Co-Immunoprecipitation and surface biotinylation of the wild-type (WT) or LL/AA mutant (Mut) OSTβ-Myc with OSTα-Flag. (**A**) The total protein extracted from transfected cells was immunoprecipitated by using anti-Myc proteinA/G agarose beads. The precipitates were separated by SDS-PAGE, and analyzed by Western blot using anti-Flag antibody to detect OSTα and anti-Myc antibody to detect OSTβ. (**B**) HEK293 cells were transiently transfected with WT or Mut OSTβ-Myc and OSTα-Flag. The OSTα and β proteins expressed on the cell surface were biotinylated and subjected to streptavidin agarose (SA) pull-down. The streptavidin agarose-bound proteins were separated by PAGE and blotted with anti-Flag or anti-Myc antibodies. The LL/AA OSTβ mutant prevents both subunits from going to the cell surface (lane #4).

Immunoprecipitation was conducted in order to determine if the LL/AA OSTβ mutant was able to interact with the OSTα subunit. HEK293 cells were transfected with a Myc-tagged OSTβand a Flag-tagged OSTα. When OSTβ-Myc was immunoprecipitated with anti-Myc agarose beads, the OSTα was efficiently precipitated only in lysates from the cells with wild type (non-mutated) OSTβ-Myc (Fig 3A, lane 1). In contrast, only a small fraction of OSTα was immunoprecipitated from lysates of cells with the LL/AA OSTβ mutant (Fig 3B. lane 2). This suggested that the physical association of the two subunits may be prevented by replacing dileucine sequences with alanines. Cell surface biotinylation was then performed to label the OSTα and β subunits that were able to reach the plasma membrane. Strepavidin bead pull-down demonstrated that when the HEK293 cells were co-transfected with both OSTα-flag and wild type OSTβ-Myc, they were both expressed on the cell surface. By contrast, mutation of L20L21 to A20A21 at the N-terminal tail of OSTβ prevents the cell surface expression of both subunits (Fig 3B). These data confirm that the extracellular dileucine motif is crucial for the interaction of OSTβ with OSTα and this interaction is necessary for plasma membrane targeting of this heteromeric transporter.

### The effect of protease inhibitors on the expression of mutant OST

**Fig 4:**
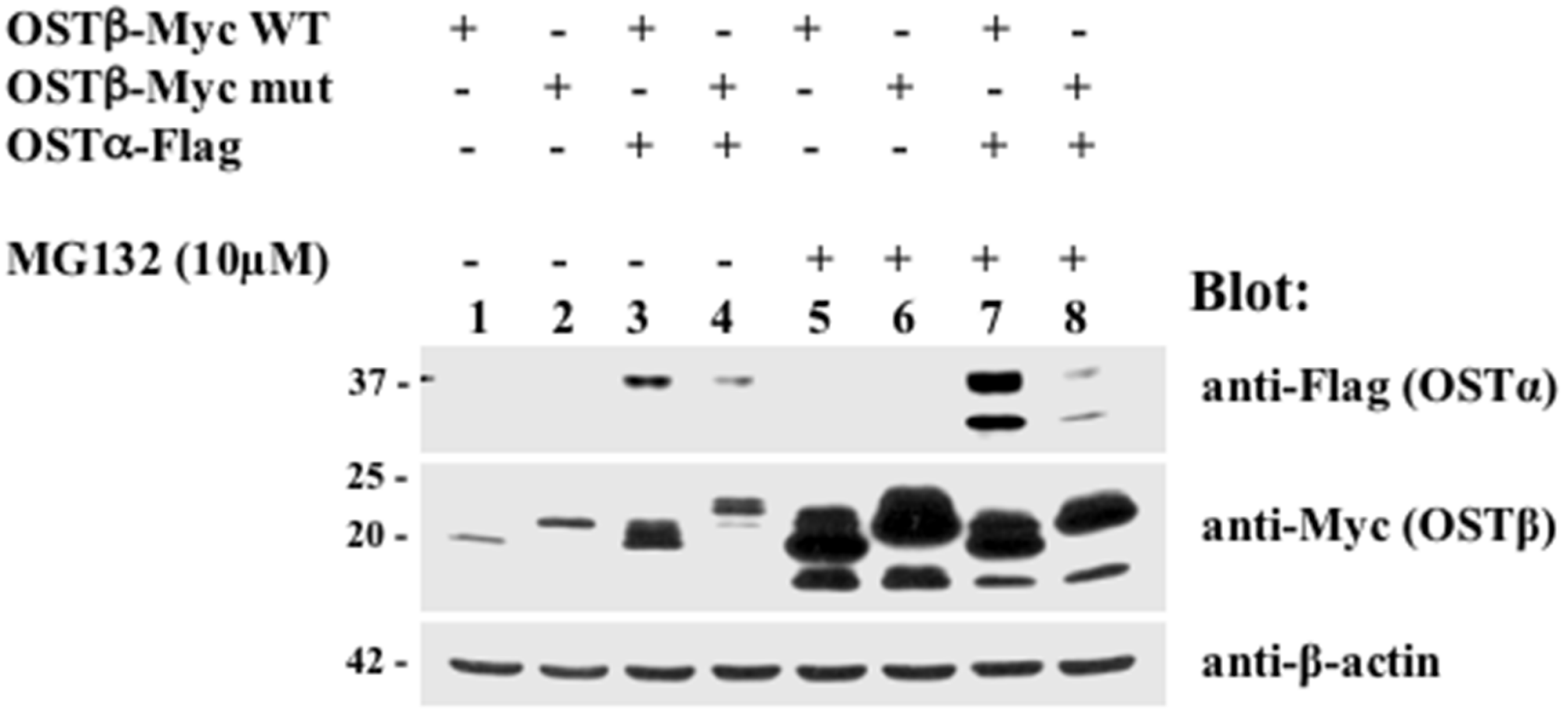
Effect of a proteasome inhibitor (MG132) on the expression of mutant OSTβ. Transfected HEK293 cells were treated with MG132 (10μM) or DMSO for 24 h. Inhibition of proteasome degradation by MG132 dramatically increased the protein levels for OSTβ in HEK293 cells. MG132 treatment did not restore the reduced OSTα levels when co-expressed with LL/AA OSTβ mutant in total cell extract in HEK293 cells (see lane 8), indicating that the inhibition of the proteasome degradation pathway by MG132 is not sufficient to stabilize the alpha subunit in the absence of the beta subunit.

To investigate a possible underlying mechanism for the significantly reduced expression of OSTα in cells transfected with the LL/AA mutant, we tested whether increased protease degradation was responsible for the loss of OSTα. There exists a possibility that preventing the interaction between subunits by replacing a dileucine sequence with a dialanine sequence in the N-terminus of OSTβ might subject the subunits to increased protein degradation. An inhibitor of proteasomal degradation, MG132, or a combination of lysosomal inhibitors, leupeptin and pepstatin, were used to treat transfected HEK293 cells for 24 hrs. Lysosomal inhibitors had no effect on OSTα-β expression (data not shown). MG132 treatment resulted in a dramatic increase in protein levels of WT and mutant OSTβ compared to untreated HEK293 cells (compare Fig 4 lanes 3 and 4 to lanes 7 and 8). And, although MG132 treatment also increased the protein level of OSTα when it was cotransfected with WT OSTβ, it showed only a minimal increase in the immature form of OSTα when it was cotransfected with mutant OSTβ in HEK293 cells (Fig 4, lane 8). This suggests that inhibition of the proteasome pathway by MG132 is not sufficient to stabilize the alpha subunit in the absence of interaction with the beta subunit. Furthermore, this confirms the results seen in Fig 2A where OSTα is undetectable if OSTβ is not present.

### Computational-based modeling of hOST beta

In order to more closely examine potential structural effects of the induced mutations, we generated a homology model of OSTβ. Computational-based analysis of the OSTβ model suggested that the LL/AA mutation would substantially alter both the protein structure and the lipophilic potential of the surface in this region of the protein. Given that the interaction between OSTα and OSTβ is thought to involve the transmembrane domain of OSTβ, alterations in this area would have the potential to affect the interaction between the alpha and beta subunit.

**Fig 5:**
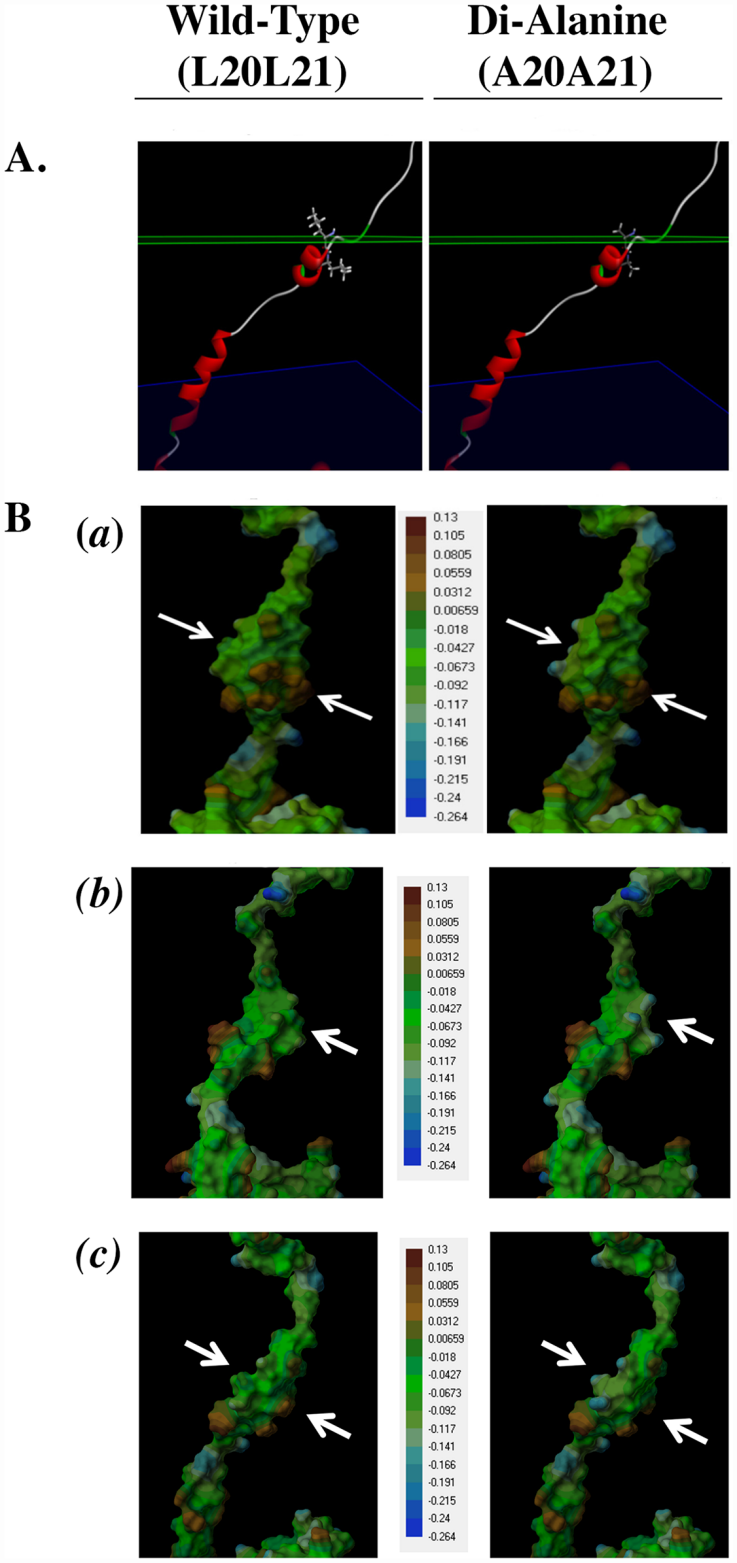
Mutation of hOSTβ (LL/AA) results in predicted alterations to protein structure and hydrophobicity. (**A**) The location of the two leucine residues near one edge of the membrane in the wild-type OSTβ (left panel) is predicted to be in a transmembrane region of the protein near the extracellular edge of the hydrophobic region of the membrane (green plane) in positions that are accessible for interaction with OSTα. Mutation of these two residues to Ala (right panel) reduces the size of the side chains at these positions. (**B)** Molecular surface representations **(*a*, *b*, *c***) of the transmembrane region of wild-type OSTβ (left panel) and the LL/AA mutant (right panel) colored based on a lipophilic potential gradient with brown designated as most hydrophobic and blue as least hydrophobic. The mutation substantially decreases the lipophilic potential in this area of the surface (arrows) in addition to altering the size and volume of this region of the protein.

To determne whether the LL/AA mutation substantially alters the structure and the surface, thus affecting the interaction with the alpha-subunit, we ran a loop refinement on the transmembrane region of the OSTβ homology model to predict the secondary structure. The two leucines in the N-terminus of OSTβ (L20L21) are right near the edge of the hydrophobic region of the membrane in positions that are accessible for interaction with OSTα. Since we could not find a crystal structure with enough similarity to OSTα to build a homology model, we can not say how these two leucines interact with the residues of OSTα, only that they are in a position to do so. Fig 5A shows the location of the residues, which are near the boundary of the hydrophobic slab of the membrane. The mutation to alanine obviously changes the size of the side chains at these positions. Fig 5B are views from several perspectives of the transmembrane region with a molecular surface added and colored based on lipophilic potential. Brown is most hydrophobic and blue is least hydrophobic. The mutation substantially decreases the lipophilic potential of the surface in this area in addition to altering the size and volume of this region of the protein. Computational-based modelling of OSTβ suggested that the LL/AA mutations substantially change both the shape and volume of the protein such that they have the potential to directly alter the steric interactions between the two subunits.

## Discussion

The organic solute transporter alpha-beta (OSTα-OSTβ, SLC51) is a unique heteromeric, facilitated organic solute transporter that is expressed on the basolateral membrane of epithelium where sterol transport is essential [20]. It is a primary transp orter in the ileum responsible for maintaining the enterohepatic circulation of bile acids by extruding bile acids from the digestive tract back to the liver via the mesenteric and portal vein. It is also up-regulated on the liver basolateral membrane during cholestasis, thereby providing an alternative route for bile acid transport from the hepatocyte. Previous studies have determined that the functional activity of this transporter requires the physical association of the two subunits, OSTα (SLC51A) and OSTβ (SLC51B), in order to exit the endoplasmic reticulum and traffic to the plasma membrane [6]. It is also known that the N-terminal region of the beta subunit is essential for the interaction of the two subunits and, thus, the functional acitivity of the transporter. Christian et al [10] suggested that the transmembrane (TM) domain of Ostβ is required for formation of a heteromer with Ostα. Sun et al [11] further demonstrated the N-terminal domain of hOSTβ contains important information for protein-protein interaction. In the present study we have focused on this N-terminal region of the beta subunit and demonstrated that subunit interaction, stability, and plasma membrane localization are greatly influenced by a dileucine motif that is conserved among multiple species.

Seward et al [1] showed functional complementation between subunits from different species, suggesting conserved domains may be responsible for protein-protein interaction. Therefore, we conducted a conserved protein domain analysis by searching the conserved domain database (CDD) of NCBI [21] using little skate Ostbeta protein sequence as a query. Conserved domain of OSTbeta family sequences as illustrated in S1 Fig are recurring units in molecular evolution and appear in protein sequences as conserved blocks of amino acid residues that have distinct functions. By analyzing the conserved protein domain family of OSTβ, we found that hydrophobic aliphatic amino acids [22], Isoleucine (I), Valine (V) and Leucine (L), and a cluster of acidic residues, Glutamate (E) or Aspartate (D), are expressed in the conserved extracellular domain in all members of the gene family (see S1 Fig). Importantly, the leucine-based residues in the N-terminal tail were evolutionarily conserved from the little skate (*Leucoraja erinacea)* to the human homolog, suggesting that the Leu residues at the N-tail of Ostβ/OSTβ might be important for the function of this heterodimer complex.

There are multiple mechanisms underlying protein interaction and cellular trafficking, some of which depend on dileucine motifs on their cytoplasmic carboxyl- and /or amino-terminal fragments [23–26]. Vergarajauregui et al. have identified two separate dileucine-type motifs that co-operate to regulate the transport of mucolipin-1 to lysosomes [27]. Kasai et al reported that in the cytoplasmic domain of syntaxin 8 two functionally distinct dileucine-based motifs act independently in its endocytic and exocytic processes [28]. These dileucine motifs, however, differ from OSTβ, where the motif is found on the extracellular portion of the protein and where it would not be anticipated to participate in internalization of the transporter. The acidic-cluster-dileucine sequence, ELLEEML, identified at the extracellular domain of OSTβ has different consensus sequence and is different from all the classical cytosolic acidic dileucine motifs implicated in endoocytic and lysosomal sorting. Protein-protein interactions are mediated by hydrophobic interactions with the leucine side chains, but also by hydrogen bonding between conserved charged amino acid residues, particularly important is highly conserved glutamate (E) residues flanking the core leucine motif regions. Proteins bind to each other at specific binding domains on each protein containing recognition of signals, like regularly-spaced leucine residues [29]. Analysis of OSTα protein sequences also revealed two linear dileucine sequences, DLLEVL and QLLRAL, within the extracellular N-tail of OST alpha family (see S2 Fig). It may be that these two subunits bind each other through their extracellular domains with the leucine-rich peptides as protein recognition motifs. This, however, must be tested further.

This study also shows that stability of the alpha subunit and the heteromeric transporter depend upon the interaction of the two subunits early in the biosynthetic pathway. The co-expression of OSTα with the LL/AA OSTβ mutant resulted in loss of expression of the alpha subunit. This is consistent with previous findings that if the heteromeric complex cannot form, the two subunits are unstable and are degraded. However, in this study using transiently transfected HEK293 cells, the beta subunit demonstrates greater stability, as seen in Figs 2 and 4 where it is still expressed in the absence of OSTα. Inhibition of proteasome degradation by treatment with MG132 increased the expression of both WT and mutant OSTβ, as well as OSTα if cotransfected with WT OSTβ. But the treatment did not increase the expression of the alpha subunit when coexpressed with the LL/AA OSTβ mutant. This is similar to the G protein-coupled receptor, γ-aminobutyric acid, which requires the expression of the GB2 subunit in order for the GB1 subunit to exit the endoplasmic reticulum [30]. The lack of stabilization by MG132 of the alpha subunit in the presence of the mutant OSTβ suggests that the degradation is very rapid and irreversible. Immunoprecipitation confirmed that OSTα did not interact efficiently with the LL/AA OSTβ mutant, although a small amount of the mature form of the alpha subunit can be seen (Fig 3A). It is difficult to determine if the lack of alpha immunoprecipitate is merely a reflection of the small amount of alpha subunit expression remaining in the cell after co-transfection with the LL/AA OSTβ mutant. However, it is clear that the mutation of the dileucines to dialanines results in rapid degradation of the alpha subunit and prevention of plasma membrane expression.

Computational-based modelling of OSTβ suggested that the LL/AA mutation substantially alters both the structure and lipophilicity of the surface which has the potential to affect the formation of a complex with the alpha subunit. In addition, this change may also impact the capacity of OSTβ to interact with the membrane as well as affect intracellular trafficking. These results were consistant with previous studies and suggest that the assembly of the heterodimer is mediated by the extracellular domain of hOSTβ with the extracellular domain of hOSTα [9], and which increases the stability of the proteins and is required for delivery of the heteromeric complex to the basolateral plasma membrane [7].

Based on the present results and previously published observations, a general model for intracellular sorting of OST is proposed. The dileucine motif on the extracellular N-terminal tail of OSTβ is critical for the association with OSTα. This association is required to convert the OSTα subunit to a mature glycosylated form and stabilize the protein. While the molecular mechanisms involved in processing this protein remain to be more clearly defined, the current findings that the dileucine signal motif at the N-terminus of OSTβ is a critical determinant of the subunits’ protein-protein interaction provides an initial step in understanding the molecular machinery that regulates this process. Understanding the mechanisms of protein interaction and trafficking of OST have important implications for how various sterol-derived molecules are handled by many cells and may provide new insights into pharmacologic targets for altering sterol homeostasis.

## Supporting Information

S1 Fig Conserved protein domain of OSTbeta superfamily. (TIF)

S2 Fig Multiple sequence alignment of OSTalpha extracellular domain. (TIF)
