# Supplementary figures and images for: A Novel Di-Leucine Motif at the N-Terminus of Human Organic Solute Transporter Beta Is Essential for Protein Association and Membrane Localization
Source: PLoS One. 2016 Jun 28;11(6):e0158269. doi: 10.1371/journal.pone.0158269 (PMC4924846; doi:10.1371/journal.pone.0158269)

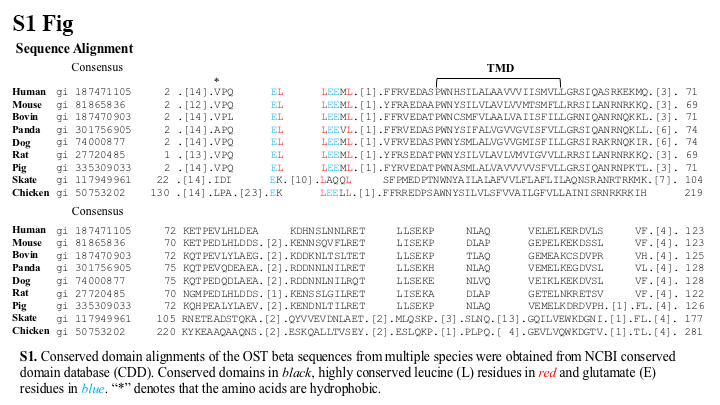

Supplement: S1 Fig — (TIF) [file pone.0158269.s001.tif]

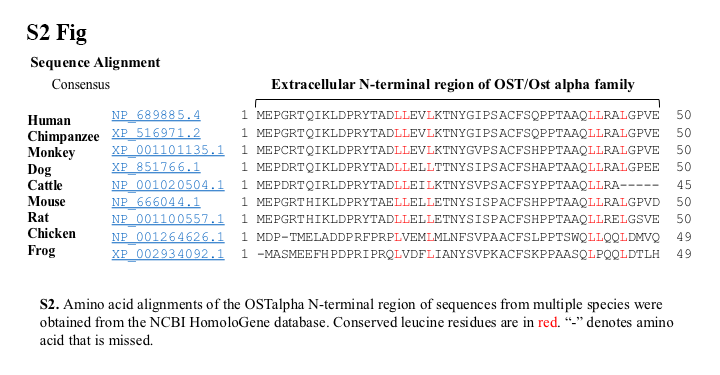

Supplement: S2 Fig — (TIF) [file pone.0158269.s002.tif]
